# Knowledge on diabetes and its related factors among the people with type 2 diabetes in Thailand: a cross-sectional study

**DOI:** 10.1186/s12889-022-14831-0

**Published:** 2022-12-16

**Authors:** Nitikorn Phoosuwan, Passakorn Ongarj, Katarina Hjelm

**Affiliations:** 1 Department of Community Health, Faculty of Public Health, Kasetsart University, 59/7 M.1 Chiangkrua, Muang, Sakon Nakhon Province Thailand; 2 Department of Public Health and Caring Sciences, Faculty of Medicine, Uppsala University, Uppsala, Sweden

**Keywords:** Diabetes Knowledge Test, Knowledge, Type 2 Diabetes

## Abstract

**Background:**

Type 2 diabetes mellitus (T2DM) is a global public health problem with complications related to knowledge guiding self-care. Limited knowledge might result in poor control of blood glucose, but there is no previous investigation measuring diabetes knowledge in people diagnosed with T2DM in Thailand. This study was aimed to investigate level of diabetes knowledge and related factors among people with T2DM in Thailand.

**Method:**

This cross-sectional study was conducted in a Northeastern province in Thailand among 276 people with T2DM, 195 women and 81 men, using a standardized self-report questionnaire, the Diabetes Knowledge Test. The data were analyzed using Pearson’s chi-square test, one-way analysis of variance, and independent samples t-test.

**Results:**

The majority of respondents had poor diabetes knowledge in all subscales; total knowledge of diabetes (96.7%), general knowledge of diabetes (71.7%), and insulin use knowledge (92.3%). There was no difference found in knowledge scores between males and females. Having finished secondary school education or higher, being employed, or having diabetes-related complications were related to increased total diabetes knowledge and general diabetes knowledge.

**Conclusion:**

The people with T2DM had poor diabetes knowledge, and those using insulin also had poor knowledge about the use of insulin.

## Background

Type 2 Diabetes mellitus (T2DM) remains a health problem worldwide, with about 463 million people with T2DM (9.3%) in 2019 [1], and the number of people with T2DM is estimated to reach 783 million in 2045 [2]. It is among the top ten causes of death in adults [2] and affects the quality of life and complications of T2DM, such as diabetes retinopathy and neurological diseases [3]. This situation has also increased rapidly in South-East Asia: from 90 million T2DM cases in 2021 to 152 million T2DM cases estimated in the next 25 years [2]. In High-Income Countries (HICs) and Middle-Income Countries (MICs), T2DM will be associated not only with lifestyles, e.g., eating habits and alcohol consumption, but also with the knowledge possessed by those who have to tackle T2DM [4]. For instance, people with T2DM in Saudi Arabia had an average level of diabetes knowledge measured with the Diabetes Knowledge Test (DKT) (66%) [5], while those in Sri Lanka had moderate and above moderate levels for diabetes knowledge (77%) [6].

Currently, in Thailand, it has been classified as an upper MIC, where it has been reported that the age-adjusted prevalence of people with T2DM is nine percent, accounting approximately 2.5 million people in Thailand suffer from T2DM, which is approximately 10% of the Thai population (10.8% in women and 8.9% in men) [7]. Thailand has a national program to screen for diabetes among people aged 35 years, and people are registered as T2DM patients and treated at a sub-district Health Promoting Hospital (SHPH) near their house [8]. A survey from the central region of Thailand found the level of knowledge of diabetes to be fair in a sample of the general Thai population (most highly educated) [9]. Another study from rural Thailand among elderly people (50–70 years) with T2DM investigated factors associated with knowledge, perception, and practice concerning self-care [10]. Gender, work and practice were shown to be related to glycemic control. However, the sample mainly included low educated (79.9%) women (69.4%). Diabetes self-management efficacy was shown to have a stronger impact on glycemic control than diabetes self-management and diabetes knowledge in a study of people with T2DM, where the majority were females (70.3%) and recruited from specialist or general hospitals in Thailand [11]. Another study focused primarily on the relationship between diabetes knowledge and self-management but also found that people with T2DM had limited knowledge about the disease (90% scored less than 22/30 points). An association between knowledge and self-management was shown [12]. Limited knowledge might result in poor control of blood glucose [13]. However, the literature review has shown no previous studies measuring the level of diabetes knowledge in people diagnosed with T2DM in Thailand.

Sakon Nakhon, the Northeastern province in Thailand, has four levels of hospitals responsible for people with T2DM (i.e., advanced hospital level, standard hospital level, medium hospital level, and small hospital level). Each hospital has primary care centers called Sub-district Health Promoting Hospitals (SHPHs). Healthcare professionals (HCPs) in the SHPH provide people with T2DM in their diabetes clinic with standard care: a trained nurse practitioner treats the people, a dietician or registered public health worker provides individual/group health education. However, there was no specific time and knowledge for health education [14]. Thus, investigating knowledge among people with T2DM in Sakon Nakhon is interesting. This study therefore aimed to assess the knowledge level and related factors for people with T2DM in the Northeastern province of Thailand.

## Methods

### Study design and setting

This cross-sectional descriptive study was conducted in Sakon Nakhon Province, Thailand from July to August 2021, using a standardized self-report questionnaire, Diabetes Knowledge Test (DKT) [15] and questions on socio-demographic background data. The study enabled researchers to gather information on the variables of interest in the people with diabetes mellitus and from the results make inferences about possible relationships between people’s socio-demographic characteristics and diabetes knowledge.

Sakon Nakhon, the Northeastern province in Thailand, is ranked the tenth poorest province in Thailand, has a mixture of residential areas (i.e., rural and urban), and has about 16,000 people registered with T2DM [16]. The data was collected in a subdistrict about 80 km from the city of Sakon Nakhon. This subdistrict has two SHPHs.

### Participants

The samples consisted of 276 adult respondents with T2DM, 195 women and 81 men, obtained through a systematic random sampling method from lists of registered people with T2DM from two SHPHs. Based on sample size calculation [17], with a significance level = 95%, *p* = 0.2 [18], *N* = 16 000, and d = 0.05, the required number of respondents was 243, a total 276 participants were included to prevent missing data in this study. The inclusion criteria were people with T2DM who (1) have been diagnosed with T2DM for ≥ one year, (2) could read and answer the questionnaire in Thai, and (3) have been registered at the selected SHPHs. The exclusion criteria reported (1) having a mental disorder or taking a psychiatric drug and (2) not being willing to participate in the study.

### Data collection

The data was collected in two SHPHs in Sakon Nakhon Province. The principal investigator (first author) asked directors of the selected SHPHs for permission to use lists of T2DM people at the diabetes clinic and collect data. Three research assistants trained by the principal investigator delivered the questionnaire to the randomly selected people with DM. Each participant completed the questionnaire voluntarily for about 20 min in their home. The questionnaires were checked for completeness by the research assistants.

### Instrument

A standardized self-reported test entitled Diabetes Knowledge Test (DKT) was used together with questions on sociodemographic characteristics.

DKT is a valid and reliable instrument [15] in order to measure general knowledge about diabetes (14 questions) and patients’ knowledge of insulin use (9 questions). Each question had multiple choices and one point if the answer was correct. A total possible score for 23 questions ranged between 0–23 points, where a score < 11 indicated poor total knowledge, 11–17 indicated average total knowledge, and ≥ 18 indicated good total knowledge [13]. A score for total general knowledge ranged between 0–17 points, where a score < 7 indicated poor general knowledge, 7–11 indicated average general knowledge, and ≥ 12 indicated good general knowledge. A score of total knowledge of insulin use ranged between 0–9 points, where a score < 5 indicated poor knowledge of insulin use, 5–7 indicated average knowledge of insulin use, and ≥ 8 indicated good knowledge of insulin use. This test was translated into Thai by three Thai English experts and back. Internal consistency was checked among 30 people with T2DM in one SHPH in Sakon Nakhon Province; Cronbach’s alpha coefficient was > 0.7, which was considered a good value for a questionnaire for data collection [19].

The questionnaire on socio-demographic characteristics included questions about gender, age, marital status, educational level, employment, religion, monthly income, and monthly expenses. The researchers had constructed this part of the questionnaire.

Three Thai researchers with a Ph.D. checked the entire questionnaire for validity. The index of Item-Objective Congruence was > 0.5 for each question, which was a satisfactory value [20].

### Ethical considerations

The Ethics Committee of Kasetsart University Chalermphrakiat Sakon Nakhon Province Campus approved the study (Reference:Kucsc.HE-62–029). It was conducted following the Declaration of Helsinki, and written informed consent was obtained [21]. The people with T2DM received both oral and written information about the study before signing a consent form in their home. The information emphasized that participation in the study was voluntary and that participants were fully entitled to withdraw at any time. None of the authors were involved in the care of the participants. Written informed consent forms were obtained from all the participants.

### Data analysis

This study used descriptive and inferential statistics. The data were analyzed using SPSS version 26 (SPSS Inc, Chicago, IL, US) for frequency, mean, standard deviation, normal distribution, chi-squared test, one-way analysis of variance (ANOVA), and independent-sample t-test. A *p*-value < 0.05 was considered statistically significant.

## Results

Most of the participants were females (70.6%), above 61 years (66%), low educated (75.4%), working as farmers (62.3%), and treated with oral medication (85.1%). See Table 1.

**Table 1:** Socio-demographic characteristics among the participants (*n* = 276)

Variables	Frequency, *n* (%)
**Gender**	
Female	195 (70.6)
Male	81 (29.4)
**Age (years)**	
≤ 50	21 (7.6)
51—60	73 (26.4)
61 – 70	116 (42.1)
≥ 71	66 (23.9)
Range = 34–87, Mean = 63.59, S.D. = 9.39	
**Marital status**	
Married	232 (84.1)
Widowed	31 (11.2)
Single	8 (2.9)
Divorced	5 (1.8)
**Educational level**	
Primary school	208 (75.4)
Secondary school	45 (16.3)
High School	15 (5.4)
Vocational school	6 (2.2)
Bachelor’s Degree	2 (0.7)
**Employment**	
Farmer	172 (62.4)
Unemployed	43 (15.6)
Daily employer	37 (13.4)
Merchant	20 (7.2)
Government officer	4 (1.4)
**Religion**	
Buddhism	276 (100.0)
**Treatment**	
Oral medication	235 (85.1)
Insulin use	28 (10.1)
Oral medication and insulin use	13 (4.7)
**Diagnosis (years)** 216 (78.3)	
1–5	79 (28.6)
6–10	54 (19.6)
> 10	83 (30.1)
Range = 1–53 $\overline{x }$=11.67 S.D. = 10.38	
**Comorbidity**	84 (30.4)
Hypertension	51 (18.5)
Dyslipidemia	23 (8.3)
Stroke	6 (2.2)
Peripheral Neuropathy	4 (1.4)
**Diabetes-related complications**	55 (19.9)
Kidney	24 (8.7)
Eye	14 (5.1)
Foot	11 (4.0)
Heart	6 (2.1)
**Monthly income (*n* = 274)**	
≤ 1,600 baht (50 dollar)	78 (28.26)
1,601 – 3,200 baht (51–100 dollar)	59 (21.38)
3,201 – 4,800 baht (101–150 dollar)	28 (10.14)
4,801 – 6,400 baht (151–200 dollar)	38 (13.77)
≥ 6,401 baht (≥ 201 dollar)	71 (25.72)
Range = 500–100,000 (15.62–3,125 dollar) $\overline{x }$ = 6,224.82 (194.52 dollar)	
S.D. = 12,457.73 (389.30 dollar)	
**Monthly expenses (*n* = 273)**	
≤ 1,600 baht (50 dollar)	103 (37.32)
1,601 – 3,200 baht (51–100 dollar)	65 (23.55)
3,201 – 4,800 baht (101–150 dollar)	33 (11.96)
4,801 – 6,400 baht (151–200 dollar)	39 (14.13)
≥ 6,401 baht (≥ 201 dollar)	33 (11.96)
Range = 400–50,000 (12.50–1,562.25 dollar) $\overline{x }$ = 3,745.05 (117.03 dollar)	
S.D. = 5,357.12 (167.41 dollar)	

*S.D*. Standard deviation

The general diabetes knowledge varied from 55.4% (question 9: For a person in reasonable control, what effect does exercise have on blood glucose?) to 13.0% (question 5: A1C is a measure of your average blood glucose level for the past). The variation in knowledge is about the same for the areas of diet (13–49%) and glycemic control (13–55%), while it is less in diabetes-related complications (29–52%) and insulin users (2–5%). See Table 2.

**Table 2:** Number and percent answered knowledge question from participants (*n* = 276)

Questions	***n* (%)**				***Answer right***
	**a**	**b**	**c**	**d**	
1. The diabetes diet is:	77 (27.9)	119 (43.1)	53 (19.2)	27 (9.8)	*119 (43.1)*
a. the way most American people eat					
*b.* *a healthy diet for most people*					
c. too high in carbohydrate for most people					
d. too high in protein for most people					
2. Which of the following is highest in carbohydrate?	79 (28.6)	63 (22.8)	79 (28.6)	55 (19.9)	*79 (28.6)*
a. Baked chicken					
b. Swiss cheese					
*c.* *Baked potato*					
d. Peanut butter					
3. Which of the following is highest in fat?	136 (49.3)	49 (17.8)	46 (16.7)	45 (16.3)	*136 (49.3)*
*a.* *Low fat (2%) milk*					
b. Orange juice					
c. Corn					
d. Honey					
4. Which of the following is a “free food”?	58 (21.0)	89 (32.2)	92 (33.3)	37 (13.4)	*37 (13.4)*
a Any unsweetened food					
b. Any food that has “fat free” on the label					
c. Any food that has “sugar free” on the label					
*d.* *Any food that has less than 20 cal per serving*					
5. A1C is a measure of your average blood glucose level for the past:	116 (42.0)	44 (15.5)	36 (13.0)	80 (29.0)	*36 (13.0)*
a. day					
b. week					
*c.* *6–12 weeks*					
d. 6 months					
6. Which is the best method for home glucose testing?	62 (22.5)	107 (38.8)	107 (38.7)	0 (0.00)	*107 (38.8)*
a. Urine testing					
*b.* *Blood testing*					
c. Both are equally good					
7. What effect does unsweetened fruit juice have on blood glucose?	91 (33.0)	128 (46.4)	57 (20.6)	0 (0.00)	*128 (46.4)*
a. Lowers it					
*b.* *Raises it*					
c. Has no effect					
8. Which should not be used to treat a low blood glucose?	107 (38.8)	57 (20.6)	79 (28.6)	33 (12.0)	*79 (28.6)*
a. 3 hard candies					
b. 1/2 cup orange juice					
*c. 1 cup diet soft drink*					
d. 1 cup skim milk					
9. For a person in good control, what effect does exercise have on blood glucose?	153 (55.4)	68 (24.6)	55 (19.9)	0 (0.00)	*153* *(55.4)*
*a.* *Lowers it*					
b. Raises it					
c. Has no effect					
10. What effect will an infection most likely have on blood glucose?	67 (24.3)	115 (41.7)	94 (34.1)	0 (0.00)	*115* *(41.7)*
a. Lowers it					
*b.* *Raises it*					
c. Has no effect					
11. The best way to take care of your feet is to:	108 (39.1)	55 (19.9)	39 (14.1)	74 (26.8)	*108* *(39.1)*
*a.* *look at and wash them each day*					
b. massage them with alcohol each day					
c. soak them for one hour each day					
d. buy shoes a size larger than usual					
12. Eating foods lower in fat decreases your risk for:	51 (18.5)	115 (41.7)	82 (29.7)	28 (10.1)	*82 (29.7)*
a. nerve disease					
b. kidney disease					
*c.* *heart disease*					
d. eye disease					
13. Numbness and tingling may be symptoms of:	97 (35.1)	145 (52.5)	22 (8.0)	12 (4.4)	*145 (52.5)*
a. kidney disease					
*b.* *nerve disease*					
c. eye disease					
d. liver disease					
14. Which of the following is usually not associated with diabetes:	39 (14.1)	57 (20.6)	53 (19.2)	127 (46.0)	*127 (46.0)*
a. vision problems					
b. kidney problems					
c. nerve problems					
*d.* *lung problems*					
15. Signs of ketoacidosis (DKA) include:	14 (5.1)	14 (5.1)	6 (2.2)	5 (1.8)	*6* *(2.2)*
a. shakiness					
b. sweating					
*c.* *vomiting*					
d. low blood glucose					
16. If you are sick with the flu, you should:	6 (2.2)	12 (4.3)	13 (4.7)	8 (2.9)	*8* *(2.9)*
a. Take less insulin					
b. Drink less liquids					
c. Eat more proteins					
*d* *Test blood glucose more often*					
17. If you have taken rapid-acting insulin, you are most likely to have a low blood glucose reaction in:	13 (4.7)	6 (2.2)	12 (4.4)	8 (2.9)	*13 (4.7)*
*a.* *Less than 2 h*					
b. 3–5 h					
c. 6–12 h					
d. More than 13 h					
18. You realize just before lunch that you forgot to take your insulin at breakfast. What should you do now?	9 (3.3)	17 (6.2)	7 (2.5)	6 (2.2)	*6 (2.2)*
a. Skip lunch to lower your blood glucose					
b. Take the insulin that you usually take at breakfast					
c. Take twice as much insulin as you usually take at breakfast					
*d.* *Check your blood glucose level to decide how much insulin to take*					
19. If you are beginning to have a low blood glucose reaction, you should:	12 (4.3)	12 (4.3)	10 (3.6)	5 (1.8)	*10 (3.6)*
a. exercise					
b. lie down and rest					
*c.* *drink some juice*					
d. take rapid-acting insulin					
20. A low blood glucose reaction may be caused by:	10 (3.6)	13 (4.7)	7 (2.5)	9 (3.3)	*10 (3.6)*
*a.* *too much insulin*					
b. too little insulin					
c. too much food					
d. too little exercise					
21. If you take your morning insulin but skip breakfast, your blood glucose level will usually:	22 (8.0)	12 (4.3)	5 (1.8)	0 (0.00)	*12 (4.4)*
a. increase					
*b.* *decrease*					
c. remain the same					
22. High blood glucose may be caused by:	13 (4.7)	8 (2.9)	10 (3.6)	8 (2.9)	*13 (4.7)*
*a.* *not enough insulin*					
b. skipping meals					
c. delaying your snack					
d. skipping your exercise					
23. A low blood glucose reaction may be caused by:	14 (5.1)	14 (5.1)	4 (1.4)	7 (2.5)	*14 (5.1)*
*a.* *heavy exercise*					
b. infection					
c. overeating					
d. not taking your insulin					

Note: Choices with italic texts are correct answers

There were 12 out of 14 questions where the participants with non-insulin use had more than 50% incorrect answers for total diabetes knowledge. The top five items incorrectly answered fell in the areas of free foods and good carbohydrate (two items), measuring and controlling blood glucose level, and complications related to food intake (one item). See Table 3.

**Table 3:** Incorrectly answered questions among general diabetes knowledge questions

Questions	*n* (%)
5. A1C is a measure of your average blood glucose level for the past:	240 (87.0)
a. day	
b. week	
*c.* *6–12 weeks*	
d. 6 months	
4. Which of the following is a “free food”?	239 (86.6)
a Any unsweetened food	
b. Any food that has “fat free” on the label	
c. Any food that has “sugar free” on the label	
*d.* *Any food that has less than 20 cal per serving*	
2. Which of the following is highest in carbohydrate?	197 (71.4)
a. Baked chicken	
b. Swiss cheese	
*c.* *Baked potato*	
d. Peanut butter	
8. Which should not be used to treat a low blood glucose?	197 (71.4)
a. 3 hard candies	
b. 1/2 cup orange juice	
*c.* *1 cup diet soft drink*	
d. 1 cup skim milk	
12. Eating foods lower in fat decreases your risk for:	194 (70.3)
a. nerve disease	
b. kidney disease	
*c.* *heart disease*	
d. eye disease	
6. Which is the best method for home glucose testing?	169 (61.2)
a. Urine testing	
*b.* *Blood testing*	
c. Both are equally good	
11. The best way to take care of your feet is to:	168 (60.9)
*a.* *look at and wash them each day*	
b. massage them with alcohol each day	
c. soak them for one hour each day	
d. buy shoes a size larger than usual	
10. What effect will an infection most likely have on blood glucose?	161 (58.3)
a. Lowers it	
*b.* *Raises it*	
c. Has no effect	
1. The diabetes diet is:	157 (56.9)
a. the way most American people eat	
*b.* *a healthy diet for most people*	
c. too high in carbohydrate for most people	
d. too high in protein for most people	
14. Which of the following is usually not associated with diabetes:	149 (54.0)
a. vision problems	
b. kidney problems	
c. nerve problems	
*d.* *lung problems*	
7. What effect does unsweetened fruit juice have on blood glucose?	148 (53.6)
a. Lowers it	
*b.* *Raises it*	
c. Has no effect	
3. Which of the following is highest in fat?	140 (50.7)
*a.* *Low fat (2%) milk*	
b. Orange juice	
c. Corn	
d. Honey	

Choices with italic texts are correct answers

Amongst participants with insulin use, there were another five items on which more than 70% respondents answered incorrectly, about “Signs of ketoacidosis (DKA),” “action when forgetting to take insulin at breakfast,” “action when sick with flu,” “action when having low blood glucose,” and “causes of low blood glucose reaction.” See Table 4.

**Table 4:** Incorrectly answered question among insulin use knowledge questions

Questions	n (%)
15. Signs of ketoacidosis (DKA) include:	33 (84.6)
a. shakiness	
b. sweating	
*c.* *vomiting*	
d. low blood glucose	
18. You realize just before lunch that you forgot to take your insulin at breakfast. What should you do now?	33 (84.6)
a. Skip lunch to lower your blood glucose	
b. Take the insulin that you usually take at breakfast	
c. Take twice as much insulin as you usually take at breakfast	
*d.* *Check your blood glucose level to decide how much insulin to take*	
16. If you are sick with the flu, you should:	31 (79.5)
a. Take less insulin	
b. Drink less liquids	
c. Eat more proteins	
*d.* *Test blood glucose more often*	
19. If you are beginning to have a low blood glucose reaction, you should:	29 (74.4)
a. exercise	
b. lie down and rest	
*c.* *drink some juice*	
d. take rapid-acting insulin	
20. A low blood glucose reaction may be caused by:	29 (74.4)
*a.* *too much insulin*	
b. too little insulin	
c. too much food	
d. too little exercise	
21. If you take your morning insulin but skip breakfast, your blood glucose level will usually:	27 (69.2)
a. increase	
*b.* *decrease*	
c. remain the same	
17. If you have taken rapid-acting insulin, you are most likely to have a low blood glucose reaction in:	26 (66.7)
*a.* *Less than 2 h*	
b. 3–5 h	
c. 6–12 h	
d. More than 13 h	
22. High blood glucose may be caused by:	26 (66.7)
*a.* *not enough insulin*	
b. skipping meals	
c. delaying your snack	
d. skipping your exercise	
23. A low blood glucose reaction may be caused by:	25 (64.1)
*a.* *heavy exercise*	
b. infection	
c. overeating	
d. not taking your insulin	

Note: Choices with italic texts are correct answers

Almost all participants had poor (score < 11) total diabetes knowledge (96.7%), while about three-fourths had poor general diabetes knowledge (71.7%). The people with T2DM using insulin had poor diabetes knowledge (92.3%). See Table 5.

**Table 5:** Knowledge about diabetes among participants using the diabetes knowledge test (DKT)

Category	Frequency *n* (%)	Description of knowledge level
**Total knowledge score (out of 23) *n* = 276**		
< 11	267 (96.7)	Poor
11–17	9 (3.3)	Average
≥ 18	0	Good
Range = 1–17 $\overline{x }$=5.59 S.D. = 2.38		
**Total knowledge score in insulin users (out of 23) *n* = 39**		
< 11	32 (82.0)	Poor
11–17	7 (18.0)	Average
≥ 18	0	Good
Range = 2–17 $\overline{x }$=7.74 S.D. = 3.13		
**General knowledge score (out of 14) *n* = 276**		
< 7	198 (71.7)	Poor
7–11	78 (28.3)	Average
≥ 12	0 (0.00)	Good
Range = 0–11 $\overline{x }$=5.26 S.D. = 2.08		
**Insulin use knowledge score (out of 9) *n* = 39**		
< 5	36 (92.3)	Poor
5–7	3 (7.7)	Average
≥ 8	0 (0.00)	Good
Range = 0–6 $\overline{x }$=2.36 S.D. = 1.51		

*S.D*. Standard deviation

There was no significant difference between males and females in total, general, and insulin use knowledge score (*p* = 0.747, *p* = 0.808 and *p* = 0.351, respectively). See Table 6.

**Table 6:** Comparison between males and females mean knowledge scores about diabetes

**Knowledge category**	**Males**	**Females**	***p*-value**
	**Means (SD)**	**Means (SD)**	
Total knowledge score (out of 23) *n* = 276	5.52 (2.53)	5.62 (2.32)	0.747
Total knowledge score (out of 23) *n* = 39	8.00 (3.71)	7.67 (3.00)	0.783
General knowledge score (out of 14) *n* = 276	5.21 (2.27)	5.28 (2.00)	0.808
Insulin use knowledge score (out of 9) *n* = 39	2.78 (2.28)	2.23 (1.22)	0.351

The results indicated that a high educational level or being employed was related to increased total diabetes knowledge score (*p* = 0.003 and *p* = 0.001, respectively) and general diabetes knowledge score (*p* = 0.005 and *p* = 0.001, respectively). Diabetes-related complications were related to an increased total diabetes knowledge score (*p* = 0.038). See Table 7.

**Table 7:** Comparison of mean knowledge scores according to the participants’ sociodemographic characteristics

Variable	Total Knowledge scores (out of 23) Mean ± SD *n* = 276	*p*-value	Total Knowledge scores in insulin users (out of 23) Mean ± SD *n* = 39	*p*-value	General knowledge score (out of 14) Mean ± SD *n* = 276	*p*-value	Insulin use knowledge score (out of 9) Mean ± SD *n* = 39	*p*-value
**Gender**		**0.747**		**0.783**		**0.808**		**0.351**
Males	5.52 ± 2.53		8.00 ± 3.71		5.21 ± 2.27		2.78 ± 2.28	
Females	5.62 ± 2.32		7.67 ± 3.00		5.28 ± 2.00		2.23 ± 1.22	
**Age (years)**		**0.811**		**0.907**		**0.678**		**0.876**
≤ 60	5.64 ± 2.40		7.33 ± 3.54		5.33 ± 2.15		2.42 ± 1.62	
≥ 61	5.57 ± 2.38		7.70 ± 3.13		5.22 ± 2.05		2.36 ± 1.51	
**Marital status**		**0.080**		**0.423**		**0.103**		**0.294**
Married	5.47 ± 2.29		7.71 ± 2.91		5.15 ± 2.03		2.42 ± 1.48	
Widowed/ Divorced	6.42 ± 2.57		8.43 ± 4.08		5.49 ± 1.96		2.43 ± 1.62	
Single	5.25 ± 3.45		4.00 ± 0.00		5.25 ± 3.45		0.00 ± 0.00	
**Educational level**		0.003*		**0.529**		0.005*		**0.496**
Primary school	4.85 ± 2.29		8.43 ± 3.46		4.64 ± 1.68		2.00 ± 1.29	
Secondary school or higher	5.83 ± 2.36		7.59 ± 3.09		5.46 ± 2.10		2.44 ± 1.56	
**Employment status**		0.001*		**0.315**		0.001*		**0.378**
Unemployed/self-employed farmer	4.61 ± 2.41		7.57 ± 2.85		4.41 ± 1.90		3.00 ± 2.16	
Employed (daily employer/merchant/government worker)	5.87 ± 2.30		9.25 ± 5.31		5.50 ± 2.07		2.28 ± 1.45	
**Diagnose (years)**		**0.591**		**0.430**		**0.713**		**0.518**
1–5	5.67 ± 2.14		9.50 ± 7.78		5.62 ± 1.96		2.00 ± 2.83	
6–10	5.93 ± 2.93		8.91 ± 4.30		5.31 ± 2.18		3.00 ± 1.41	
> 10	6.06 ± 2.34		7.74 ± 1.90		5.52 ± 2.20		2.37 ± 1.61	
**Diabetes-related complications**		0.038*		**0.992**		**0.337**		**0.713**
Yes	6.22 ± 2.76		7.75 ± 3.45		5.51 ± 2.15		2.25 ± 1.48	
No	5.45 ± 2.27		7.74 ± 2.96		5.20 ± 2.06		2.43 ± 1.56	

*p < 0.05

## Discussion

This study is unique as it investigated levels of diabetes knowledge and related factors among people with T2DM in Thailand. The main results showed that the participants diagnosed with T2DM had poor diabetes knowledge for total knowledge (96.7%), general knowledge (71.7%), and insulin use knowledge (92.3%). There was no difference in knowledge scores between genders but finishing secondary school education or higher, being employed, and having diabetes-related complications were related to having more diabetes knowledge.

Limited knowledge is often related to self-care management among T2DM people [13, 22], and diabetes knowledge might be at a low level among persons with T2DM in both HICs and MICS, e.g., about two-thirds of Saudi Arabian people with T2DM were found to have a moderate level of diabetes knowledge in a previous study [5]. Our result is consistent with previous studies in Asia; it revealed that 30% of Vietnamese with T2DM had a low level of diabetes knowledge [23], and 75% of Chinese with T2DM [24]. However, none of these studies used the same instrument for measuring knowledge. The Chinese study [24] was a pilot survey to assess nutrition knowledge as the primary focus. In contrast, a previous study from Thailand [12] investigated the relationship between diabetes knowledge and diabetes self-management among T2DM people using a self-answered questionnaire, the level of diabetes knowledge was also low (most T2DM people had scored lower than 22/30 points). Diabetes management should include special considerations that are culturally acceptable for Thais with T2DM [25]. In addition, the participants in this study emphasized that they lacked knowledge about how to select low glucose diets, to manage their blood glucose level, including signs when they had high or low blood glucose. An educational program for T2DM people focusing on the nutritional and eating program might increase knowledge and self-care management among T2DM people [3, 24], e.g., a diabetes self-management education [26].

The employed participants seemed to have higher diabetes knowledge scores than self-employed farmers or unemployed. Thai farmers need to seek information and exchange ideas to have a better standard of living, but they need to work every day from 6 a.m. to 6 p.m. [27]. They might not have time to gain diabetes knowledge elsewhere. Due to age and having a low level of education, those that are unemployed and aged over 60 years may find it difficult to seek knowledge or understand information regarding diabetes [13, 28]. In addition, the participants who finished primary school had less diabetes knowledge than those who finished secondary school or a higher level. The people with T2DM and low educational level (finished primary school) might have difficulty with health-seeking behaviors, such as access to and ability to use digital searching [28], which leads to less chance of obtaining information about diabetes. The people with T2DM visited a diabetes clinic for regular follow-up, and received only a few minutes of diabetes education from HCPs [14]. Therefore, to improve diabetes literacy and knowledge, it is necessary to give education to patients and staff in diabetes. Especially, giving diabetes education in an appropriate time frame and with specific diabetes materials by trained HCPs needs to be developed [13, 22, 29].

In this study, having diabetes-related complications was related to increased diabetes knowledge. In Thailand, there is an appointment for the registered T2DM people to receive regular treatment and they often receive information about diabetes care from SHPHs they visit [8]. Participation in a diabetes class might be a beneficial program to increase diabetes knowledge for people with T2DM [13]. Therefore, regular follow-ups and management for the people with T2DM should be offered, and regularly attending a quality diabetes class should be promoted.

### Strengths and limitations

We used a standardized questionnaire, the DKT [15], and tested the questionnaire with reasonable content validity and internal consistency [19, 20]. Sample size calculation and sample randomization were applied. Therefore, our findings could represent the people with T2DM in Sakon Nakhon Province and might be generalized to people in other provinces in Thailand having similar characteristics.

This study was a cross-sectional descriptive study, which could only describe the relationship between socio-demographic characteristics and diabetes knowledge. Using a questionnaire implies having structured questions, and the contents are thus predetermined and do not give room for a deeper understanding of the respondents’ beliefs and thoughts. On the other hand, it enabled researchers to gather information on the variables of interest in the people with T2DM and make inferences about possible relationships between socio-demographic characteristics of people and diabetes knowledge.

A limitation of the study was that it was conducted in the Northeastern province, ranked the fifteenth poorest province in Thailand [30], thus limiting generalizability to other parts of the country which are similar in characteristics. Nevertheless, it is an important starting point. The population in the study showed that there was a high number of females (70.6%); thus, the representativeness can be questioned, but on the other hand, T2DM has been shown to be more prevalent in women than men (55% vs. 45%) [7].

A qualitative study might investigate diabetes knowledge and related factors and contribute to a clearer understanding. This study had only some participants with insulin use. Hence, a future study focusing on T2DM people with insulin treatment might be of interest.

## Conclusions

The people with T2DM had poor total diabetes knowledge, general diabetes knowledge, and insulin use knowledge. Secondary school education or higher education, employment with permanent income, and having diabetes-related complications were related to increasing total diabetes knowledge and general diabetes knowledge. Diabetes self-management education focusing on a nutritional and eating program is needed for people with T2DM. Repeated diabetes knowledge education is recommended to provide diabetes knowledge and self-care management knowledge.

## Data Availability

The datasets used and/or analyzed during the current study available from the corresponding author on reasonable request.

## Abbreviations

DKT: Diabetes knowledge test
HCP: Healthcare professionals
HIC: High-income country
MIC: Middle-income country
SHPH: Sub-district health promoting hospital
T2DM: Type 2 Diabetes Mellitus
